# Athlete Performance Monitoring in Anti-Doping

**DOI:** 10.3389/fphys.2018.00232

**Published:** 2018-03-23

**Authors:** James Hopker, Yorck O. Schumacher, Matthew Fedoruk, Jakob Mørkeberg, Stéphane Bermon, Sergei Iljukov, Reid Aikin, Pierre-Edouard Sottas

**Affiliations:** ^1^Endurance Research Group, University of Kent, Chatham, United Kingdom; ^2^Aspetar Orthopaedic and Sports Medicine Hospital, Doha, Qatar; ^3^United States Anti-Doping Agency, Colorado Springs, CO, United States; ^4^Anti-Doping Denmark, Brøndby, Denmark; ^5^LMAHESS Nice, France and Monaco Institute of Sports Medicine and Surgery, University Cote d'Azur, Monaco, France; ^6^KIHU—Research Institute for Olympic Sports, Jyvaskyla, Finland; ^7^World Anti-Doping Agency, Lausanne, Switzerland

**Keywords:** anti-doping, monitoring, target testing, data analytics, competition results

The use of information technology within sport has significantly increased over recent years. These data and information have the potential to make a significant impact on sporting performance, and the nature of its related sciences too. For example, the retrospective analysis of sporting performance data affords the possibility to identify the impact of various technological advances and rule changes on world record performances in sports such as, javelin throwing (+95% over 76 years), pole vault (+86% in 94 years), and 1-h track cycling (+221% in 111 years; Haake, 2009). Similarly, such types of longitudinal data analysis may also be useful from an anti-doping perspective. In this regard, it has previously been shown that yearly world best performances increase with the emergence of new potent doping agents, such as anabolic steroids or EPO (Schumacher and Pottgiesser, 2009). Conversely, when new anti-doping tests are implemented, overall world best performances decrease as the effects of certain performance enhancing drugs become detectable, and are therefore avoided by athletes (Schumacher and Pottgiesser, 2009). These findings raise the possibility that performance monitoring can be useful for anti-doping efforts.

As the aim of any doping regime is to improve sporting performance, it has been suggested performance data, in the form of an Athlete Performance Module (APM), may be useful in strengthening the sensitivity and applicability of the current Athlete Biological Passport (ABP) in the fight against doping in sports (Schumacher and Pottgiesser, 2009). However, there is a general view that performance biometrics alone are not sufficient evidence to establish doping, and as such, cannot demonstrate the use of a prohibited substance in accordance with the World Anti-Doping Code (Article 2.2). Even though sudden increases in performance can be caused by reasons other than doping (e.g., improved training or nutritional strategies), such observations may nevertheless provide worthwhile information in order to trigger targeted anti-doping tests of specific athletes (Iljukov et al., 2018). In addition, whilst not sufficient to convict an athlete for doping, an atypical individual performance profile may also be useful as corroborative evidence in, for example, an ABP case. However, to date, the use of performance data for anti-doping purposes by National Anti-Doping Organizations (NADOs) and International Federations (IFs) remains low.

## The need for robust performance data

Even though access to performance data is growing, data quality, and accessibility remain important barriers to overcome, especially as differences exist across sports and levels of competition. For example, performance results and rankings are often available for top-level international events, but less so at more regional and national levels. This is important for the use of performance data in anti-doping given that mostly a *change* in performance, rather than the absolute level of performance may be an indicator of doping. Therefore, the availability of data for longitudinal tracking of athletes over the course of their careers is critical to identify an individual's performance progress. As such, a key component of this longitudinal monitoring is to be able to differentiate between “normal” increases in performance caused by maturation and training, from an “unnatural” improvement caused by doping. A lack of available data concerning performance development and variability therefore makes it difficult to interpret an individual athlete's performance changes, and whether they are “realistic” or not. From the limited studies that have investigated the within-athlete variability of performance, it is apparent that elite athlete individual variation of performance over a season in so-called Centimeter-Gram-Second (CGS) sports appears to be relatively small, with for example, the coefficient of variation ranging from 1.1 to 1.4% (90% CI: 1.0–1.6%) in track and field athletics (Malcata and Hopkins, 2014). Moreover, the between season variability in performance also appears relatively stable, for example coefficient of variations as low as 1% (90% CI: 0.9–1.1%) have been reported for elite rowing athletes (Smith and Hopkins, 2011). Thus, the priority for the development of an APM should be the collection of sport specific performance data, together with the identification of potential confounding factors affecting this data (e.g., pacing, tactics, environmental conditions etc.). Research is required in order to develop an evidence basis for “normal” seasonal variations and longitudinal changes in performance across sports. Moreover, there is a need to identify the typical rates of performances increases in sports as athletes' transition across junior categories to the elite rankings, as well as the inevitable decline in performance with aging (Berthelot et al., 2012).

The actual performance metric(s) that should be used in an APM is yet to be determined. While the use of general performance metrics such as rank finishing position may offer advantages of broad applicability to the majority of sports, sport specific performance metrics (SSPM) are anticipated to increase the ability to identify changes in performance that provide more resolution for anti-doping purposes. In CGS sports (e.g., track and field disciplines or weightlifting, where the results are directly measurable in these units), the competitive results themselves can effectively be used as a performance metric. In non-CGS sports, the development of specific metrics may need to be further explored. For example, in combat sports, the number of hits and take-downs per round could be a sport-specific performance metric, which changes over a fighter's career and may be associated with performance improvements in the sport. Furthermore, the performance of an athlete in relation to their competitors might also be a valuable tool (e.g., mass start cycling) where the monitoring of race results via ranked finishing position (e.g., UCI ranking points; Filipas et al., 2017), or the use of an Elo-rating system (Elo, 2008) such as that currently used in chess, could be viable alternatives to assess performance trends. Thus, even though there is a need to develop SSPM, it could be argued that the APM should be generic, enabling its application across a range of different sports (e.g., Olympic sports). Whatever the SSPM, it is important that the data has a low risk of being manipulated by the athlete, or any of their support team.

## Development of an APM

### Performance modeling

In order to provide a proof of concept and establish the usefulness of performance monitoring in the identification of potential doping in sports, retrospective analysis of available existing performance data is a good starting point. However, this analysis may only currently be possible in a small number of sports due to the availability of sufficient performance data. There is a need for IFs, and the anti-doping community more generally, to collect and archive sport performance data that may be used to establish SSPM and meaningful thresholds for an APM.

Analysis of “big data” contained within performance repositories has the potential to increase our understanding of athlete performance and how it evolves over an athlete's career. However, appropriately “unlocking” this information and being able to effectively distinguish performance changes due to doping, from natural performance evolution, represents a significant analytical challenge. Data mining may provide a method by which raw data is translated into information by analyzing and interpreting its patterns, particularly where some kind of predictive ability is possible. Thus, a retrospective trawl of existing data repositories for a particular sport might be used to establish typical performance trends within a large cohort of athletes. With this information, it may then be possible to distinguish between career trajectories of presumed “clean” athletes vs. those previously sanctioned for doping offenses. Any retrospective analysis must account for the possibility of biased competition data due to dopers not being caught, which is highly likely due to the possible high prevalence of doping in elite sport (Ulrich et al., 2017). Moreover, establishing the accuracy of large datasets retrospectively is often impractical, especially if 3rd party websites are used as sources of information, where the competition results are reported to accurately reflect the “official” results. Using a data mining approach also often requires a certain level of computerized automation, and so it is imperative that data is checked for structural errors, filtered, and cleaned prior to analysis. Therefore, the complexity of the data and its preparation for analysis represents a significant cost in terms of resource (human and technology), and is a challenge for implementing such an approach.

### Use of micro-technology

Micro-technology is being increasingly utilized for day-to-day training and recovery monitoring in order to track physiological performance parameters. These portable devices (e.g., GPS watch, power meters, activity monitors etc…) are being increasing used by athletes and coach to monitor exercise during both training and competition in order to provide a detailed analysis of performance patterns at a microscopic level (Esteve-Lanao et al., 2005; Sanders et al., 2017). The utilization of such data could provide useful information from an anti-doping perspective. For example, human physiology imposes an unavoidable law on exercise performance and the duration it can be sustained, which are inversely related. Thus, the higher the exercise intensity, the shorter the duration of exercise. Therefore, using micro-technology it is possible to create an individualized athlete signature of their habitual training patterns, and assess the resultant changes within their physiological capacities during competition. This type of data may allow the determination of distinct patterns of training and resultant changes in performance, which could flag any greater than expected progressions that could be suspicious. As such, combining this type of performance biometric data with physiology biometrics may complement the ABP. Performance data collected directly, for example from a runner's GPS watch or a cyclist's power meter, might also serve to explain seemingly unusual changes in performance. In this regard, historical training and competition data, combined with doping control test histories and normal biological passports, might be used to legitimize an athlete's performance that within the isolated context of one particular race/event, seems extraordinary. However, there are currently several important considerations using such an approach, including the validity and reliability of the micro-technology used to collect the data, as well as the data itself (Menaspà and Abbiss, 2017). Moreover, as outlined above (see section Performance Modeling), careful consideration would need to be provided to the analysis of the data collected in order to extract meaningful information.

### Risk prediction

It may also be possible to include performance data as part of a wider risk prediction to identify athletes who are more likely to be involved in doping. For example, athletes who demonstrate large and unexpected increases in performance over time, both in relation to their previous performance trajectory, and that of their peers, might be flagged as being “high risk” for doping. Indeed, Marclay et al. (2013) suggest a wide range of information should be encompassed within the interpretation of data for anti-doping purposes, integrating not just analytical chemical results and longitudinal monitoring of biomarkers, but also information on athlete whereabouts, social media presence, athlete competitive level, potential financial rewards, social networks, and competitive performance data. This information could therefore be used to rank athletes according to their probability of being involved in doping, and might allow a more strategic approach to the allocation of the most “high risk” athletes to registered testing pools.

## The outlook for athlete performance monitoring within anti-doping

Despite the perceived burden on IFs to generate databases of SSPM, the authors feel that the inclusion of sport-related performance data in the form of an APM will enhance the ability of anti-doping authorities to make more informed decisions on assigning athletes to registered testing pools. As such, the APM may also allow a more effective distribution of anti-doping testing resources to target those athletes who demonstrate potentially abnormal increases in performance, or are deemed more at risk of doping. It is envisaged that the APM could be integrated into the athlete monitoring tool-kit of any NADO or IF, and thus allow the combination of unique aspects of information from athletes in order to enhance current anti-doping efforts.

## Author contributions

All authors were involved in the conceptualization of the article and its contents. JH drafted the article, and all authors reviewed, edited, and approved the final version.

### Conflict of interest statement

The authors declare that the research was conducted in the absence of any commercial or financial relationships that could be construed as a potential conflict of interest.
